# Gene Expression, Network Analysis, and Drug Discovery of Neurofibromatosis Type 2-Associated Vestibular Schwannomas Based on Bioinformatics Analysis

**DOI:** 10.1155/2020/5976465

**Published:** 2020-07-15

**Authors:** Qiao Huang, Si-Jia Zhai, Xing-Wei Liao, Yu-Chao Liu, Shi-Hua Yin

**Affiliations:** Department of Otolaryngology & Head and Neck Surgery, The Second Affiliated Hospital of Guangxi Medical University, Nanning 530007, China

## Abstract

Neurofibromatosis Type 2- (NF2-) associated vestibular schwannomas (VSs) are histologically benign tumors. This study aimed to determine disease-related genes, pathways, and potential therapeutic drugs associated with NF2-VSs using the bioinformatics method. Microarray data of GSE108524 were downloaded from the Gene Expression Omnibus (GEO) database, and differentially expressed genes (DEGs) were screened using GEO2R. The functional enrichment and pathway enrichment of DEGs were performed using Gene Ontology (GO) and Kyoto Encyclopedia of Genes Genomes (KEGG). Furthermore, the STRING and Cytoscape were used to analyze the protein-protein interaction (PPI) network of all differentially expressed genes and identify hub genes. Finally, the enriched gene sets belonging to the identified pathways were queried against the Drug-Gene Interaction database to find drug candidates for topical use in NF2-associated VSs. A total of 542 DEGs were identified, including 13 upregulated and 329 downregulated genes, which were mainly enriched in terms of focal adhesion, PI3K-Akt signaling pathway, ECM-receptor interaction, Toll-like receptor signaling pathway, Rap1 signaling pathway, and regulation of actin cytoskeleton. 28 hub genes were identified based on the subset of PPI network, and 31 drugs were selected based on the Drug-Gene Interaction database. Drug discovery using bioinformatics methods facilitates the identification of existing or potential therapeutic drugs to improve NF2 treatment.

## 1. Introduction

VSs, also known as acoustic neuromas, are histologically benign tumors originating from the eighth nerve. NF2 is a rare autosomal dominant inherited disorder tumor caused by deletion or loss-of-function mutations in the NF2 gene encoding merlin [1]. The main characteristic of NF2-associated VSs is the bilateral schwannomas of the vestibular nerve, which leads to sensorineural hearing loss, facial paralysis, vestibular dysfunction, brainstem compression, and even death [2]. Despite their benign nature, NF2-associated VSs have poor prognosis prone to recurrence, and there are no curative treatments. At present, the primary treatments are follow-up observation, microsurgery, and radiosurgery which are not always effective and sometimes cause neurological deficits [3]. Patients with hearing loss sometimes accept the otolaryngology surgery and require improving or saving hearing. With the targeted molecular therapies becoming increasingly common, drug therapy has gradually become possible. Therefore, it is urgently required to determine effective drug targets for NF2-associated VSs therapies. The present study aimed to determine disease-related genes, pathways, and potential targeted therapeutic drugs associated with NF2-associated VSs using the bioinformatics method.

## 2. Materials and Methods

### 2.1. Microarray Datasets

The gene expression profile GSE108524 of the NF2-associated VSs and normal nerve groups was obtained from the NCBI GEO database. These microarray data were based on GPL17586 Platform [HTA-2_0] Affymetrix Human Transcriptome Array 2.0 [transcript (gene) version], including 17 NF2-associated VSs tissues and 4 normal nerves.

### 2.2. Identification of DEGs

GEO2R (http://www.ncbi.nlm.nih.gov/geo/geo2r/), a web tool based on the analysis of variance or *t*-test, was used to identify DEGs between NF2-associated VSs tissues and normal nerves. The DEGs were identified as the genes with |log FC| ≥ 1.5 and adj. *P* < 0.05.

### 2.3. Functional and Pathway Enrichment Analysis of DEGs

The Database for Annotation, Visualization, and Integrated Discovery (DAVID) (Version 6.8, https://david.ncifcrf.gov/) was used to perform GO and KEGG pathway enrichment analysis of DEGs. GO analysis contains biological process (BP), cellular component (CC), and molecular function (MF). GO term with the criterion of *P* < 0.05 and false discovery rate (FDR) < 0.05 and KEGG pathway analysis with the criterion of *P* < 0.05 were considered statistically significant.

### 2.4. Protein-Protein Interaction (PPI) Network Analysis

We submitted DEGs in Search Tool for the Retrieval of Interacting Genes database (STRING, http://www.string-db.org/) to screen the PPI pairs with a combined score of ≥0.4 and visualized the interaction using Cytoscape software (Version 3.7.0.). Finally, CentiScaPe and Molecular Complex Detection (MCODE), a Cytoscape plugin, were utilized to screen PPI network key genes. The default parameters of MCODE were used: degree cutoff ≥2, node score cutoff ≥0.2, k-score  ≥ 2, and maximum depth = 100.

### 2.5. Drug-Gene Interaction Analysis

To better identify potential targeted therapeutic drugs for NF2-associated VSs, the hub genes were mapped onto the Drug-Gene Interaction database (DGIdb; http://www.dgidb.org) to obtain potential drug target genes and potential NF2-associated VSs treatment drugs. Visualization of the drug-gene interaction was generated using Cytoscape software (Version 3.7.0.). In addition, ClinicalTrials.gov (https://clinicaltrials.gov) was used to identify whether drugs have been previously investigated or are being currently tested in clinical trials.

### 2.6. Human NF2-Associated VSs Specimens

Human NF2-associated VSs tissues with the matched normal adjacent specimens were obtained from the Second Hospital of Guangxi Medical University. This study was approved by the Ethics Committee of the Second Hospital of Guangxi Medical University.

### 2.7. Quantitative PCR (qPCR)

Reverse transcription was carried out using SYBR premix EX Taq (Takara, Japan), and SYBR Premix Ex Taq II (Takara) was used for qPCR. We used several sequences: EGFR forward primer 5′-CTACAACCCCACCACGTACC-3′ and reverse primer 5′-CGCACTTCTTACACTTGCGG-3′; GAPDH forward primer 5′-CTTCGCTCTCTGCTCCTCCTGTTCG-3′ and reverse primer 5′-ACCAGGCGCCCAATACGACCAAAT-3. The results were calculated using the 2^−ΔΔCt^ method.

### 2.8. Statistical Analysis

Statistical analysis was conducted by SPSS 20.0 software. The statistical significance between groups was determined using a two-tailed Student's *t*-test. Values of *P* < 0.05 were considered to indicate statistically significant differences.

## 3. Results

### 3.1. Identification of DEGs

**Figure 1:**
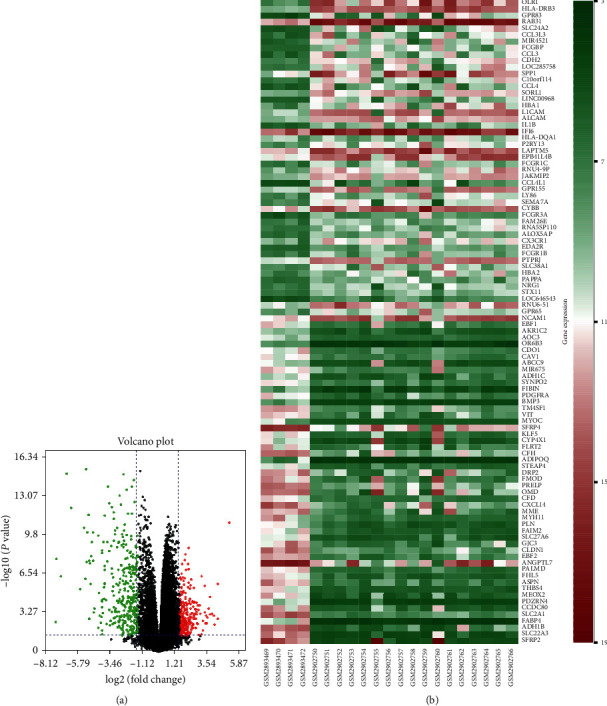
(a) DEGs were selected by volcano plot filtering (|fold change | ≥  1.5 and adj. *P* < 0.05). (b) The heat map of DEGs in NF2-associated VSs (top 100 upregulated and downregulated genes). Green represents a downregulated expression, and red indicates an upregulated level.

A total of 542 DEGs, including 13 upregulated and 329 downregulated genes, were identified by comparing 17 NF2-associated VSs tissues and 4 normal nerves from GSE108524. The heat map and volcano plot showed these DEGs (Figure 1).

### 3.2. Functional Annotation and Pathway Enrichment Analysis of DEGs

GO functional annotation revealed that the DEGs were significantly enriched in BP terms including cell adhesion, inflammatory response, immune response, signal transduction, positive regulation of protein kinase B signaling, positive regulation of ERK1 and ERK2 cascade, and positive regulation of GTPase activity. In addition, the CC terms mainly showed plasma membrane, extracellular exosome, extracellular region, extracellular matrix, and membrane raft. MF enrichment indicated heparin binding and integrin binding (Table 1). Furthermore, KEGG pathway enrichment analysis revealed focal adhesion, PI3K-Akt signaling pathway, ECM-receptor interaction, Toll-like receptor signaling pathway, Rap1 signaling pathway, and regulation of actin cytoskeleton (Table 2).

### 3.3. PPI Network Analysis

**Figure 2:**
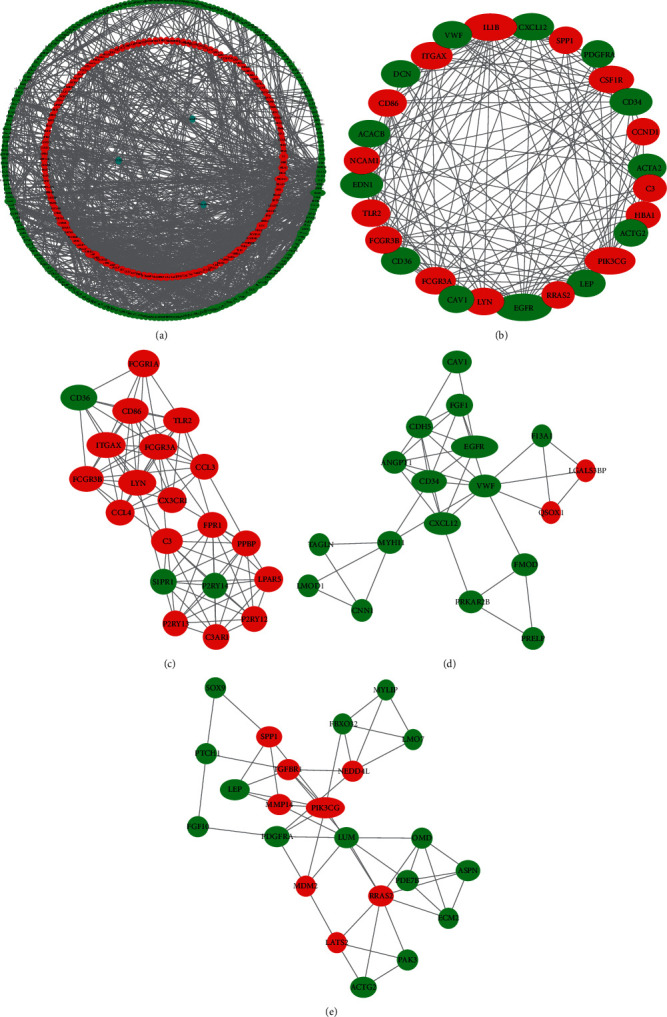
(a) The PPI network of DEGs. (b) The hub genes with connectivity degree ≥20. (c) Module 1. (d) Module 2. (e) Module 3. Green represents a downregulated expression, and red indicates an upregulated level.

In total, we made the PPI network of 369 nodes and 1,322 edges, based on the STRING database (Figure 2(a)). We identified 28 hub genes with connectivity degree ≥20 (Figure 2(b), Table 3). Then, using MCODE, three modules with scores >4.5 and a number of nodes >18 were selected. Module 1 with a score of 9.368 consisted of 20 nodes and 89 edges (Figure 2(c)), module 2 with a score of 4.588 comprised 18 nodes and 39 edges (Figure 2(d)), and module 3 with a score of 4.455 comprised 23 nodes and 49 edges (Figure 2(e)).

### 3.4. Drug-Gene Interaction Analysis

**Figure 3:**
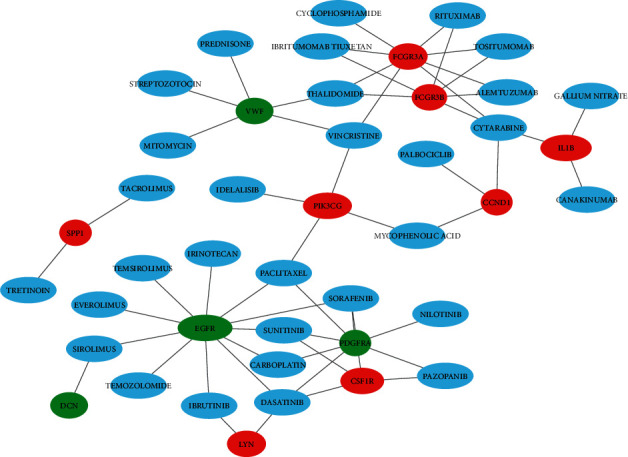
Drug-gene interactions of hub genes.

Based on the DGIdb, we use the 28 hub genes to screen for drug-gene interactions, which revealed that 31 drugs associated with 12 key genes may be potential NF2 treatment drugs (Figure 3). Based on ClinicalTrials.gov, we found that nilotinib was previously investigated for Phase 2 of growing VSs treatment and everolimus is being used in Phase 2 of the NF2 treatment study.

### 3.5. mRNA Expression Levels of EGFR

**Figure 4:**
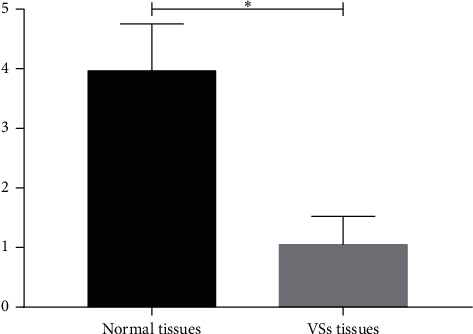
The mRNA expression levels of EGFR (^*∗*^*P* < 0.05).

qPCR analysis verified EGFR mRNA underexpression levels in the NF2-associated VSs tissues (Figure 4).

## 4. Discussion

In this study, we found that the 28 hub genes had been insufficiently studied or not studied at all in VSs, 12 of which may be target genes for potential NF2 treatment drugs. Among these genes, IL1B, PIK3CG, CSF1R, LYN, FCGR3A, FCGR3B, SPP1, and CCND1 were upregulated in NF2-associated VSs, while EGFR, DCN, VWF, and PDGFRA were downregulated. Then, LYN, FCGR3A, and FCGR3B are involved in “module 1” of the subnetwork, in which GO functional annotation is enriched in inflammatory response and immune response, and KEGG pathway enrichment analysis is enriched in staphylococcus aureus infection, phagosome, and osteoclast differentiation. EGFR and VWF are involved in “module 2,” which is enriched in focal adhesion and PI3K-Akt signaling pathway. PIK3CG and SPP1 are involved in “module 3,” which is also enriched in focal adhesion and PI3K-Akt signaling pathway.

We found that upregulated genes PIK3CG, CSF1R, SPP1, and CCND1 and downregulated genes EGFR and VWF were significantly enriched in PI3K-Akt signaling pathway involved in VSs development, which can increase schwannoma cell proliferation, survival, and cell-matrix adhesion acting [4–6]. That may be the cause of poor prognosis in NF2-associated VSs. The drugs that inhibit the PI3K-Akt signaling pathway may be a potential therapeutic strategy for NF2 by antitumor activity against NF2-related tumor cells.

Merlin, a tumor suppressor, is constantly inactivated in NF2-associated VSs. SPP1, also known as osteopontin (OPN), is a secreted, integrin-binding phosphoprotein. OPN had been insufficiently studied in VSs, while elevated OPN is a utility of some tumors progression and metastasis, suggesting a poor prognosis, such as breast cancer [7]. Morrow et al. study [7] revealed that OPN-initiated signaling induced Akt-mediated phosphorylation and degradation of merlin in breast cancer cells; it was reported for the first time that OPN is involved in merlin protein degradation. We showed that SPP1 is upregulated in NF2-associated VSs, consistent with the result of Torres-Martin et al. [8]. SPP1 may be a biomarker of NF2-associated VSs, whose interaction with merlin has not been reported in NF2-associated VSs. Furthermore, we found that drugs associated with SPP1, including tacrolimus and tretinoin, may be potential therapeutic agents for NF2-associated VSs, which require a one-step study. Tacrolimus, a powerful immunosuppressant, significantly increased OPN mRNA and protein expression from kidney tissue and renal cells, which may contribute to nephrotoxicity inducing [9]. However, tacrolimus used to treat autoimmunity blocks IL2 production and is used for active rheumatoid arthritis [10] and lupus nephritis [11]. Based on functional annotation and pathway enrichment analysis of DEGs, inflammatory response, immune response, melanoma, and rheumatoid arthritis may be connected with NF2-associated VSs development. Therefore, tacrolimus may be used for NF2-associated VSs treatment.

In our study, CCND1 involved in apoptosis and cell cycle control, a key cell cycle regulatory protein, was upregulated in NF2-associated VSs, which is consistent with previous studies [12, 13]. Elevated CCND1 is known to suggest poor prognosis in many cancers, such as colorectal cancer [14], breast cancer [15], and multiple myeloma [16, 17]. We found drugs associated with CCND1, including palbociclib and mycophenolic acid, which had not been studied in VSs. Palbociclib, a cyclin-dependent kinase 4 and 6 (CDK4/6) inhibitor, prolongs progression-free survival among patients with advanced estrogen receptor-positive and HER2-negative breast cancer [18, 19]. Mycophenolic acid, an immunosuppressant, can inhibit proliferation and induce apoptosis in cancer cells, which may be caused by inhibition of upregulation of CCND1 and the PI3K/AKT/mTOR pathway [20]. Very interestingly, CCND1 was also upregulated in NF2-associated VSs and was significantly enriched in the PI3K-Akt signaling pathway in this study. Thus, palbociclib and mycophenolic acid may inhibit the growth of NF2-associated VSs.

In contrast to SPP1 and CCND1, EGFR was downregulated in NF2-associated VSs, in agreement with the results of Torres-Martin et al. [8], but contrary to those of Yi et al. [21]. At present, the efficacy of EGFR inhibitors in acoustic neuroma treatment is not ideal yet, which may be related to EGFR downregulated in some patients.

In conclusion, with the present analysis, we identified 28 drugs not yet tested in NF2-associated VSs. Tacrolimus, palbociclib, and mycophenolic acid may be candidate drugs. SPP1 and CCND1 may be potential targeted genes in NF2-associated VSs. PI3K-Akt signaling pathway may be involved in VSs development.

## Figures and Tables

**Table 1:** GO analysis of differentially expressed genes.

Category	Term	Count	*P* value	FDR
BP	GO:0007155 ∼ cell adhesion	38	<0.001	<0.001
BP	GO:0006954 ∼ inflammatory response	34	<0.001	<0.001
BP	GO:0006955 ∼ immune response	32	<0.001	<0.001
BP	GO:0007165 ∼ signal transduction	58	<0.001	0.0054
BP	GO:0051897 ∼ positive regulation of protein kinase B signaling	12	<0.001	0.0203
BP	GO:0070374 ∼ positive regulation of ERK1 and ERK2 cascade	17	<0.001	0.0261
BP	GO:0043547 ∼ positive regulation of GTPase activity	34	<0.001	0.0265
BP	GO:0030198 ∼ extracellular matrix organization	18	<0.001	0.0285
BP	GO:0030335 ∼ positive regulation of cell migration	17	<0.001	0.0488
CC	GO:0005615 ∼ extracellular space	87	<0.001	<0.001
CC	GO:0005887 ∼ integral component of plasma membrane	88	<0.001	<0.001
CC	GO:0005886 ∼ plasma membrane	177	<0.001	<0.001
CC	GO:0005578 ∼ proteinaceous extracellular matrix	31	<0.001	<0.001
CC	GO:0009986 ∼ cell surface	42	<0.001	<0.001
CC	GO:0070062 ∼ extracellular exosome	121	<0.001	<0.001
CC	GO:0005576 ∼ extracellular region	81	<0.001	<0.001
CC	GO:0031012 ∼ extracellular matrix	28	<0.001	<0.001
CC	GO:0009897 ∼ external side of plasma membrane	21	<0.001	<0.001
CC	GO:0045121 ∼ membrane raft	20	<0.001	0.0020
CC	GO:0016021 ∼ integral component of membrane	179	<0.001	0.0020
CC	GO:0005925 ∼ focal adhesion	27	<0.001	0.0122
CC	GO:0045202 ∼ synapse	17	<0.001	0.0224
MF	GO:0008201 ∼ heparin binding	20	<0.001	<0.001
MF	GO:0005178 ∼ integrin binding	14	<0.001	0.0041

**Table 2:** KEGG pathway analysis of differentially expressed genes.

Term	Count	*P* value	Genes
hsa05150: staphylococcus aureus infection	13	<0.001	C3AR1, C3, HLA-DRB3, FPR1, C1R, C1S, HLA-DQA1, FCGR1A, CFH, FCGR3A, CFD, SELPLG, FCGR3B
hsa04145: phagosome	19	<0.001	MRC1, NOS1, OLR1, C3, TUBB2A, HLA-DRB3, TLR2, HLA-C, C1R, TLR6, HLA-DQA1, CYBB, CD36, FCGR1A, COMP, CLEC7A, FCGR3A, FCGR3B, THBS4
hsa04514: cell adhesion molecules (CAMs)	16	<0.001	CLDN19, HLA-DRB3, HLA-C, L1CAM, NLGN3, CDH2, HLA-DQA1, CDH5, ALCAM, NCAM1, CD86, CD34, ITGA8, CLDN1, CD4, SELPLG
hsa04640: hematopoietic cell lineage	12	<0.001	CR1, CD37, CD36, CD34, HLA-DRB3, FCGR1A, MME, IL1B, CD4, ANPEP, CSF2RA, CSF1R
hsa05144: malaria	9	<0.001	CR1, CD36, COMP, TLR2, IL1B, HBA2, HBA1, HBB, THBS4
hsa04610: complement and coagulation cascades	10	<0.001	C3AR1, VWF, CR1, C3, F13A1, CFH, TFPI, C1R, C1S, CFD
hsa04510: focal adhesion	17	0.0017	PIK3CG, EGFR, CAV1, TNXB, TNC, FLNB, MYL9, VWF, CCND1, PAK3, CCND2, COMP, ITGA8, COL6A3, PDGFRA, SPP1, THBS4
hsa04060: cytokine-cytokine receptor interaction	18	0.0022	EGFR, CCL3, TGFBR1, LIFR, EDA2R, CCL4L1, CCL4, CXCL12, IL17RA, LEP, PPBP, CXCL14, CCL3L3, CX3CR1, PDGFRA, IL1B, CSF2RA, CSF1R
hsa05140: leishmaniasis	9	0.0027	CR1, C3, HLA-DRB3, FCGR1A, TLR2, IL1B, FCGR3A, FCGR3B, HLA-DQA1
hsa04151: PI3K-akt signaling pathway	23	0.0033	EGFR, PIK3CG, FGF7, TNXB, TNC, TLR2, FGF10, IRS1, DDIT4, VWF, CCND1, LPAR5, CCND2, COMP, ITGA8, COL6A3, PDGFRA, MDM2, ANGPT1, FGF1, SPP1, THBS4, CSF1R
hsa00350: tyrosine metabolism	6	0.0063	MAOA, AOX1, ADH1C, ADH1B, ADH1A, AOC3
hsa03320: PPAR signaling pathway	8	0.0075	LPL, CD36, OLR1, PLIN1, SLC27A6, FABP4, ACADL, ADIPOQ
hsa04512: ECM-receptor interaction	9	0.0094	VWF, CD36, TNXB, COMP, TNC, ITGA8, COL6A3, SPP1, THBS4
hsa04620: Toll-like receptor signaling pathway	10	0.0100	PIK3CG, CD86, CCL3, CCL3L3, TLR2, CCL4L1, IL1B, TLR6, CCL4, SPP1
hsa05323: rheumatoid arthritis	9	0.0101	CD86, CCL3, HLA-DRB3, CCL3L3, TLR2, IL1B, ANGPT1, CXCL12, HLA-DQA1
hsa05218: melanoma	8	0.0103	PIK3CG, EGFR, CCND1, FGF7, PDGFRA, MDM2, FGF10, FGF1
hsa04015: Rap1 signaling pathway	15	0.0126	FYB, PIK3CG, EGFR, FGF7, FPR1, FGF10, APBB1IP, DOCK4, PLCB4, LPAR5, RASGRP3, PDGFRA, ANGPT1, FGF1, CSF1R
hsa05416: viral myocarditis	7	0.0126	CAV1, CD86, CCND1, HLA-DRB3, SGCD, HLA-C, HLA-DQA1
hsa04730: long-term depression	7	0.0160	PLA2G4A, PLCB4, NOS1, GRIA2, LYN, GUCY1A2, GUCY1B3
hsa05206: microRNAs in cancer	18	0.0176	EGFR, TNXB, CYP1B1, TNC, MIRLET7F1, MIR99A, ZEB1, MIR222, MIR221, IRS1, DDIT4, NOTCH3, CCND1, CCND2, PDGFRA, MDM2, MARCKS, MIR181B2
hsa05152: tuberculosis	13	0.0178	MRC1, CR1, ITGAX, C3, FCGR1A, HLA-DRB3, TLR2, IL1B, CLEC7A, FCGR3A, TLR6, FCGR3B, HLA-DQA1
hsa05205: proteoglycans in cancer	14	0.0191	PIK3CG, EGFR, CAV1, LUM, FZD1, TLR2, DCN, FLNB, CCND1, CBLB, GPC3, RRAS2, MDM2, PTCH1
hsa05143: African trypanosomiasis	5	0.0250	PLCB4, IL1B, HBA2, HBA1, HBB
hsa05332: graft-versus-host disease	5	0.0250	CD86, HLA-DRB3, IL1B, HLA-C, HLA-DQA1
hsa05142: Chagas disease (American trypanosomiasis)	9	0.0254	PIK3CG, CCL3, PLCB4, C3, TGFBR1, CCL3L3, TLR2, IL1B, TLR6
hsa05200: pathways in cancer	22	0.0267	EGFR, PIK3CG, FGF7, TGFBR1, FZD1, RUNX1T1, FGF10, CXCL12, CBLB, CCND1, PLCB4, LPAR5, RASGRP3, SLC2A1, PDGFRA, MDM2, PTCH1, PTCH2, HHIP, FGF1, CSF2RA, CSF1R
hsa04810: regulation of actin cytoskeleton	14	0.0282	PIK3CG, EGFR, FGF7, FGF10, NCKAP1L, MYL9, ARPC1B, ITGAX, CHRM3, PAK3, ITGA8, RRAS2, PDGFRA, FGF1
hsa04380: osteoclast differentiation	10	0.0349	PIK3CG, CYBB, FCGR1A, TGFBR1, IL1B, FCGR3A, TREM2, FCGR3B, CSF1R, BLNK

**Table 3:** 28 hub genes with connectivity degree ≥20.

Number	Gene	Degree of connectivity	Regulation
1	EGFR	59	Down
2	IL1B	53	Up
3	PIK3CG	49	Up
4	CSF1R	40	Up
5	CXCL12	39	Down
6	CD34	36	Down
7	EDN1	36	Down
8	ITGAX	34	Up
9	ACACB	34	Down
10	LYN	32	Up
11	FCGR3A	32	Up
12	DCN	30	Down
13	CD36	30	Down
14	VWF	30	Down
15	CD86	29	Up
16	TLR2	29	Up
17	ACTA2	29	Down
18	LEP	29	Down
19	FCGR3B	26	Up
20	NCAM1	25	Up
21	CAV1	24	Down
22	HBA1	23	Up
23	ACTG2	22	Down
24	SPP1	21	Up
25	C3	21	Up
26	PDGFRA	20	Down
27	CCND1	20	Up
28	RRAS2	20	Up

## Data Availability

The data used to support the findings of this study are available from the corresponding author upon request.
